# Long-term impact of parenting-related leave policies on adolescents’ well-being: a systematic review of quasi-experiments

**DOI:** 10.1093/eurpub/ckad228

**Published:** 2024-01-09

**Authors:** Hande Tugrul, David Stuckler, Arnstein Aassve

**Affiliations:** Department of Social and Political Sciences, Bocconi University, Milano, Italy; Department of Social and Political Sciences, Carlo F. Dondena Centre for Research on Social Dynamics and Public Policy, Bocconi University, Milano, Italy; Department of Social and Political Sciences, Carlo F. Dondena Centre for Research on Social Dynamics and Public Policy, Bocconi University, Milano, Italy

## Abstract

**Background:**

Parenting-related leave policies have gained increasing endorsement across Organisation for Economic Co-operation and Development (OECD) countries in recent decades. Previous reviews have focused on the short-term impacts and found predominantly positive effects on children. Although there is a growing interest in the long-term impact during adolescence and young adulthood, a comprehensive assessment of this aspect is currently lacking.

**Methods:**

We systematically reviewed studies from three electronic databases (Scopus, Web of Science and PubMed), which used quasi-experimental design and examined policies legislating the introduction or expansion of parenting-related leave policies in North America or Europe. We looked at studies focused on well-being beyond the age of 12 and analyzed the findings across different domains of well-being: health, education and labour market outcomes.

**Results:**

The quasi-experimental evidence is rather limited. The introduction of leave policies or gender-specific quotas produces substantial benefits in the long run. Further, maternal socioeconomic and educational background appears to play a substantial moderating role between leave and adolescents’ well-being. Adolescents with mothers who have higher levels of education have demonstrated a more pronounced advantage from the extended time spent together, thereby accentuating pre-existing disparities.

**Conclusions:**

Though the expansion of already long leaves might not generate significant outcomes, the introduction of leave policies or gender-specific quotas produces substantial long-term benefits. This evidence entails considerable policy implications for countries that lack a national leave policy or offer only short durations of paid leave, such as the USA.

## Introduction

Parenting-related leaves, referring to maternity, paternity and parental leave, are now endorsed across most OECD countries. Initially legislated to meet the childrearing needs of working women and to regulate their re-entry into the job market, these policies gained prominence in response to the increasing participation of women in the workforce. Presently, these leaves transcend their original objectives, as their multifaceted influence on the well-being of children and families has established them as integral components of family policies throughout OECD countries except for the USA.^1^

Maternity leaves, deeply rooted in traditional societal norms, establish mothers as the primary caregivers. Following changing gender role attitudes and the shift towards involving both parents in caregiving responsibilities, more gender-neutral and inclusive parenting-related leave policies have emerged. Allowing either parent to take time off, parental leave promotes flexibility in the household. While parental leave often permits parents to determine how they will allocate the leave period, certain gender-specific policies pioneered in Norway assign non-transferable periods to fathers, known as ‘father quotas’, to foster fathers’ leave uptake. Besides maternity and parental leave, most European countries offer short-duration, job-protected father-specific paternity leave to support fathers’ bonding with newborns and promote gender equality.

One of the primary motivations of parenting-related leave policies is their potential to influence children’s well-being. Parenting-related leave policies reduce parental labour supply after childbirth, thereby increasing parents’ time investments in children.^2–5^ Besides, they directly impact available financial resources that can be directed toward children’s development.^6^ Recognizing the early years of a child's life as a critical period for holistic growth,^7^ parenting-related leaves present a unique opportunity to nurture positive emotional, behavioural, cognitive, and physical development. The benefits of a nurturing environment will potentially radiate through adolescence and young adulthood, developing their academic performance, social relationships, labour market outcomes and physical and mental health. Though the initial foundation laid during early years can continue to influence individuals as they progress through life, the impact of parenting-related leaves may become less pronounced during adolescence and early adulthood, as these stages are characterized by increasing independence and a broader range of factors influencing one’s well-being.

Though a consensus exists regarding the short-term benefits of parenting-related leaves on children,^8–10^ their long-term impacts are disputed. Considerable research examines policy effects on adolescent and young adult physical and mental health, education and labour outcomes, but evidence varies significantly among countries. This variability stems from historical policy heterogeneity and remaining differences today.^11^ There are also empirical challenges coming from the fact that long-term causal effects are difficult to implement due to the scarcity of longitudinal data. Experimental studies are scarce due to ethical concerns, so scholars typically resort to quasi-experimental designs which involve comparing individuals exposed to an intervention with those who were not. While quasi-experiments enable retrospective analysis and offer random-like assignments, they provide lower bounds for policy effects and may have limited external validity. To the best of our knowledge, there has not yet been a systematic review summarizing existing evidence. Against this backdrop, this study makes an essential contribution by undertaking the first systematic review using well-established approaches that are rigorous and transparent. We systematically reviewed studies that adopted a quasi-experimental approach to analyze parenting-related leave policies’ long-term impact on adolescents and young adults. We synthesized the results against three domains of well-being: health, education and labour market outcomes.

## Methods

We performed the systematic review following the PRISMA guidelines.^12^ Our search strategy, inclusion criteria and analysis methods were specified in advance and documented in a protocol registered with the International Prospective Register of Systematic Reviews.

### Search strategy

We searched Scopus, Web of Science and PubMed on 20 April 2022, for peer-reviewed articles using high-quality research designs which attempt to ascertain the causal effect of parenting-related leave policies on the well-being during adolescence and young adulthood.

To operationalize the search terms for parenting-related leave policies, we drew upon keyword variants previously validated in systematic reviews.^13^^,^^14^ Further, we identified four domains of long-term well-being and specified search terms by drawing upon at least one validated systematic review per domain: education,^15–17^ physical and mental health,^14^ labour market outcomes^18^^,^^19^ and deviant behaviour.^14^ To review exclusively quasi-experimental research, we added a series of terms specific to study design. The final search string is reported in Supplementary material S2.

**Figure 1:**
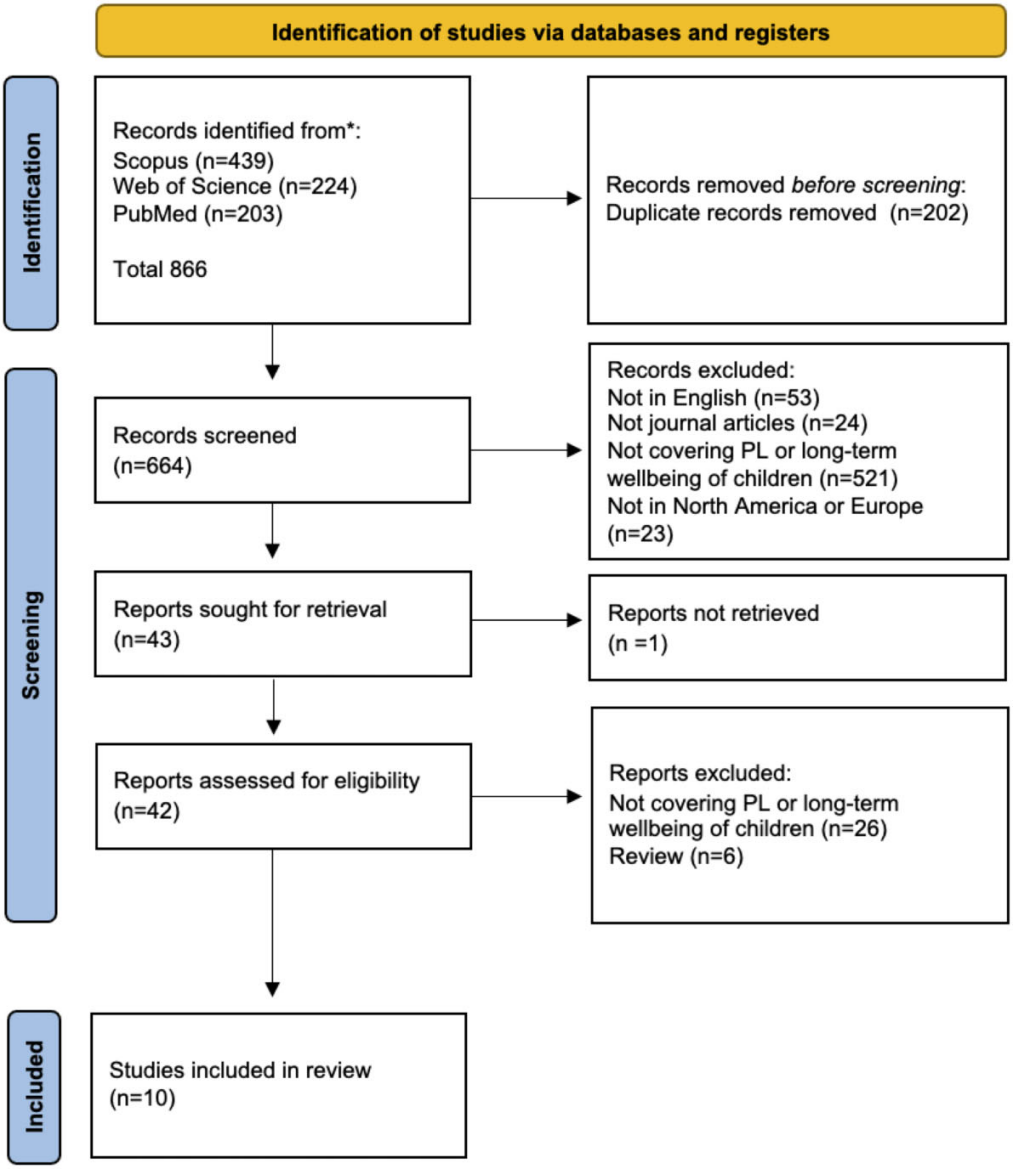
PRISMA flow diagram

Our initial search identified 439 articles in Scopus, 224 in Web of Science and 203 in PubMed. Of these 866 articles, which were imported to Zotero reference management software,^20^ 202 were identified as duplicates, leaving a total of 664 for screening and eligibility stages. Figure 1 shows the PRISMA flow diagram for study inclusion.

### Eligibility criteria

We applied a series of inclusion and exclusion criteria regarding participants, intervention, comparison, outcomes, and study design (PICOS). The full details of the search and associated PICOS are available in Supplementary material S1. We included the articles if they were: (i) written in English, (ii) published in a peer-reviewed journal, (iii) employed a quasi-experimental design, (iv) analyzed the policies legislating the introduction or expansion of maternal or parental or paternal leave, (v) considered well-being above 12 years of age as the outcome. They were excluded if the study focused on countries outside North America or Europe, intending to review cases from countries with comparable socioeconomic status.

Of 664 articles screened, we excluded 53 for not being in English, 24 for not being peer-reviewed articles, 521 for not covering parenting-related leave policies or the long-term well-being and 23 for focusing on countries outside North America or Europe. This left us with 43 articles for retrieval. We were unable to find the full text for 1 article, resulting in 42 articles for eligibility. Upon reviewing the complete text, we excluded another 26 articles for not covering parenting-related leave or the long-term well-being and 6 for being identified as reviews. This left us with a total of 10 articles for the final review.

### Extraction and analysis

For the 10 included studies, we extracted data using a preestablished format. We collected the following elements in the full text: country and year in which the intervention took place, type of the leave policy and the intervention under analysis, duration of the leave before and after the intervention, well-being outcomes, overall results, heterogeneity of the results according to subgroups, the impact on parental outcomes (the channels of influence outlined) and the study design. We further grouped the studies according to their outcome domains and composed a data extraction table for each. As some studies touch upon multiple outcomes and thus deliver multiple data points, we included them in more than one table (tables 1–3).

**Table 1:** Studies on the long-term impact of parenting-related leave on the health outcomes during adolescence or young adulthood

Study	Country	Year/s	Leave type	Intervention type	Duration before/after	Health outcome/s	Average results	Heterogeneity in results	Impact on parental outcomes	Study design
Ahammer et al. (2020)	Austria	1974	Maternal (Prenatal)	Duration extension of paid leave	From 6 to 8 weeks	Aggregate spending in the outpatient sector and aggregate days spent in hospital between 25-40 years of age	No significant effect	N/A	Fertility: no significant effect on the quantum or tempo of subsequent fertility Long-term health and mortality: no significant impact within 20 years after the birth. Only marginally significant effect within 40 years.	Regression discontinuity design and instrumental variable and difference-in-differences
Fabel (2021)	Germany	1979	Maternal	Duration extension of paid leave	From 2 to 6 months	Hospital admissions and diagnosis between 17 and 35 years of age	Positive	Hospitalization: stronger among men and above 20. Diagnosis: results are mainly driven by mental and behavioural disorders	Due to a lack of data, the study could only assess the impacts on parental outcomes.	Difference-in-differences
Ginja et al (2020)	Sweden	1986	Parental	Duration extension of Speed Premium Policy	From 24 to 30 months	Hospital admissions at ages 10 and 14	No significant effect	A significant effect on the likelihood of being admitted to the hospital at age 14 for the firstborn child of 0.6% points	Delay in return to work: increase in maternal time across all socioeconomic classes. Short- and long-term income: on average no significant impact. But there is compensation behaviour of fathers in top one-third households which is small and short lives (dissipates when the younger child reaches 2 years old)	Regression discontinuity design

**Table 2:** Studies on the long-term impact of parenting-related leave on the education outcomes during adolescence or young adulthood

Study	Country	Year/s	Leave type	Intervention type	Duration before/after	Education outcome/s	Average results	Heterogeneity in results	Impact on parental outcomes	Study design
Carneiro et al (2015)	Norway	1977	Maternal	Introduction of paid and duration extension of unpaid leave	Paid leave: from 0 to 4 months. Unpaid leave: from 3 to 12 months.	High school dropout rate and college attendance by age 30	Positive	Positive effects stronger for children from less educated households, females, and later-born children	Delay in return to work: not directly measured. Assume high leave take-up for paid leave based on reforms in the 1990s. Assume low leave take-up for unpaid leave based on the insignificant effect of the reform on short-term maternal income. Short- and long-term income: no impact of the reform on maternal labour supply and income in the short and long term (5 years after the birth).	Regression discontinuity design and difference-in-differences
Cools et al (2015)	Norway	1992; 1993	Parental and paternal	Duration extension of paid parental leave in 1992 and introduction of paternal quota in 1993	1992 and 1993 reforms provided an additional 3 weeks of parental leave, which parents could share freely. 1993 reform also introduced an additional 4-week paternal quota (lost if not taken by the father)	Exam score at the end of lower secondary (around age 16)	Mixed. No effect of 1992 reform. Positive effect of 1993 reform.	For 1993 reform: positive impact higher in families where the father has at least as high education as the mother	Delay in return to work: no effect of the paternal quota on fathers’ work hours. Short- and long-term income: no effect of the paternal quota on paternal yearly earnings and maternal labour market attachment. The extension of general leave in 1992 had a positive impact on maternal earnings and propensity to work at least part-time. Fertility: no effect of the paternal quota on family stability or fertility	Regression discontinuity design and difference-in-differences
Dahl et al (2016)	Norway	1987, 1988, 1989, 1990, 1991, 1992	Maternal	Duration extension of paid leave	1987: from 18 to 20 weeks 1988: from 20 to 22 weeks 1989: from 22 to 24 weeks 1990: from 24 to 28 weeks 1991: from 28 to 32 weeks 1992: from 32 to 35 weeks	Exam score at the end of lower secondary (around age 16) and high school graduation by age 20	No effect	N/A	Short- and long-term income: no or little effect on parental earnings and participation in the labour market in the short or long run. Fertility: no or little impact on completed fertility, marriage, or divorce	Regression discontinuity design
Danzer and Lavy (2018)	Austria	1990	Parental	Duration extension of paid leave	From 12 to 24 months.	PISA score at age 15	No effect	The impact was significant and positive for children of highly educated mothers, especially boys. In contrast, the effect was significant and negative for children of lower-educated mothers.	Delay in return to work: high leave take-up. Short- and long-term income: no significant differences in labour supply or earnings after five or ten years. Fertility: positive and significant impact on fertility outcomes in the short (3 years) and medium run (10 years). High-wage mothers did not change fertility rates, but increased intervals and low-wage mothers increased the total number of births in the next ten years.	Regression discontinuity design and difference-in-differences
Dustmann and Schönberg (2012)	Germany	1979, 1986, 1992	Maternal	Duration extension of paid and unpaid leave	1979: paid leaves from 2 to 6 months. 1986: paid leaves from 6 to 10 months 1992: 18 months of paid extended with 18 months of unpaid leave	1979: track choice and average years of schooling (sample between 28-32 years old) 1986: graduation from the high track by age 20 1992: track choice at age 14	Mixed. No effect for 1979 and 1986. The negative effect for 1992.	N/A	Delay in return to work: leave take-up decreases in each. Low SES mothers respond more to benefits (paid leaves). Short- and long-term income: short-run income reduction in 1979 and 1986; larger loss in 1992. No impact over long-term labour market outcomes	Regression discontinuity design and difference-in-differences
Ginja et al (2020)	Sweden	1986	Parental	Duration extension of Speed Premium Policy	From 24 to 30 months	GPA in ninth grade and college attendance by 24 years old	Positive	Positive effects are stronger for later-born children and children of mothers from higher socioeconomic status	Delay in return to work: increase in maternal time across all socioeconomic classes. Short- and long- term income: on average no significant impact. But there is compensating behaviour of fathers in the top one-third households, which is small and short-lived (not significant—dissipates when the younger child reaches 2 years old)	Regression discontinuity design
Liu and Skans (2010)	Sweden	1988	Parental	Duration extension of paid leave	From 12 to 15 months	Compulsory school grades and national tests are taken at 16	No effect	Positive effect for children of mothers from higher socioeconomic status	Short- and long-term income: no effects on mothers’ future labour earnings. Fertility: no impact on fertility and parental separations. Health: no effect on maternal mental health	Difference-in-differences
Rasmussen (2010)	Denmark	1984	Parental	Duration extension of paid leave	From 14 to 20 weeks	High school enrolment up to age 21 and high school GPA (if completed high school)	No effect	N/A	Delay in return to work: high female labour force participation and leave take-up rates. Short- and long-term income: slightly positive effect on short-term labour market outcome (mothers' incomes and career opportunities). No significant impact on long-term labour market outcomes	Regression discontinuity design

**Table 3:** Studies on the long-term impact of parenting-related leave on the labour market outcomes during young adulthood

Study	Country	Year/s	Leave t type	Intervention type	Duration before/after	Labour market outcome/s	Average results	Heterogeneity in results	Impact on parental outcomes	Study design
Ahammer et al (2020)	Austria	1974	Maternal (prenatal)	Duration extension of paid leave	From 6 to 8 weeks	Employment, occupation, and wages up to age 40	No effect	N/A	Fertility: no significant effect on the quantum or tempo of subsequent fertility. Long-term health and mortality: no significant impact within 20 years after the birth. Only marginally significant effect within 40 years after the birth	Regression discontinuity design and instrumental variable
Carneiro et al (2015)	Norway	1977	Maternal	Introduction of paid and extension of unpaid leave	Paid leave: from 0 to 4 months. Unpaid leave: from 3 to 12 months.	Wages (sample between 25 and 33 years of age)	Positive	Positive effect stronger for children from less educated households, for males, and later-born children	Delay in return to work: not directly measured. Assume high leave take-up for paid leave based on reforms in the 1990s. Assume low leave take-up for unpaid leave based on the insignificant effect of the reform on short-term maternal income. Short- and long-term income: no impact of the reform on maternal labour supply and income in the short and long term (5 years after the birth)	Regression discontinuity design and difference-in-differences
Dustmann and Schönberg (2012)	Germany	1979	Maternal	Duration extension of paid leave	From 2 to 6 months	Wages (sample between 28 and 32 years of age)	No effect	N/A	Delay in return to work: leave take-up decreases in each. Low SES mothers respond more to benefits (paid leaves). Short- and long-term income: short-run income reduction in ‘79 and ‘86 because benefits did not compensate; larger loss in ‘92. No impact over long-term labour market outcomes	Regression discontinuity design and difference-in-differences

## Results

### Health

We identified three studies analyzing the long-term impact of parenting-related leave on the health outcomes during adolescence or young adulthood using a quasi-experimental design.^21–23^ All papers exploited the extension of existing paid leaves. The overall results demonstrated only weak evidence; specifically, only one paper, Fabel,^23^ found a significant positive impact of extended paid maternity leave (Table 1).

Turning first to the papers which did not detect an effect, Ahammer et al.^21^ evaluated the 1974 reform in Austria, which extended the mandatory paid prenatal maternity leave from 6 to 8 weeks. Using data on the spending in the outpatient sector and aggregate days spent in the hospital between 25 and 40 ages, the authors applied a regression discontinuity design to compare those exposed with the new reform with those not. The estimates failed to detect a significant effect, which authors attributed to Austria’s pre-existing healthcare system allowing pregnant women to take sick leave with a medical certificate.

Another paper that failed to detect any significant effect is Ginja et al.’s study^22^ exploiting the 1986 reform in Sweden, which extended the so-called Speed Premium rule of the paid parental leave system from 24 to 30 months. The rule instructs the extension of parental leave benefits if parents have two births within a predetermined number of months. Thus, the aim is to incentivize closely spaced births and provide the same benefits for the new child. Yet, it unintentionally increases the older child’s time with the mother while holding the new child’s time constant. The authors used regression discontinuity design to compare hospital admissions from 10 to 14 years old. The overall estimates, in which both siblings are included, did not reveal any significant impacts during adolescence.

Unlike the studies mentioned above, Fabel’s^23^ estimates disclosed a positive impact on overall long-term health outcomes. Using a differences-in-differences approach, he studies the effects of the 1979 extension of paid maternal leave in the Federal Republic of Germany from 2 to 6 months on hospital admissions and diagnoses between ages 16 and 35. He found that individuals born after the expansion had fewer hospital admissions and were less likely to be diagnosed with mental and behavioural disorders. Furthermore, positive results were stronger among males, and the results were mainly driven by the urban areas where female labour force participation is higher. He suggested that the positive impact was due to a shift from informal care, primarily provided by grandparents or non-professionals, to parental care, believed to offer higher quality.

### Education

We identified eight studies that employed a quasi-experimental design to analyze the long-term impact of parenting-related leave on the educational outcomes during adolescence or young adulthood.^22^^,^^24–30^ Two papers failed to see any significant effect,^25^^,^^29^ two found no impact on average but observed significant positive effects among subgroups,^24^^,^^30^ two found mixed results^26^^,^^28^ and two identified positive impact (Table 2).^22^^,^^27^

Turning first to the papers which did not detect an overall effect, a study by Rasmussen^25^ focused on the duration extension of paid parental leave from 14 to 20 weeks in 1984 in Denmark. By applying regression discontinuity design, he compared the high school enrolment and GPA of adolescents born shortly before and after the reform. The author could not detect a measurable effect, suggesting it might be due to the initial leave duration (over 3 months) and a minor extension (6 weeks). Additionally, with high-quality, publicly subsidized day care available in Denmark during the 1980s, the author argued that the reform inadvertently reduced the quality of received day care by promoting a shift towards parental care. Likewise, adopting the same study design, Dahl et al.^29^ evaluated a series of extensions of paid maternity leave in Norway between 1987 and 1992. They concluded that none of the reforms impacted exam scores at the end of lower secondary or high school.

Danzer and Lavy^30^ and Liu and Skans^24^ also observed that the duration extension of paid parental leave did not generate any significant effect on average. Yet, interestingly, both papers identified heterogeneity in their results when the analyses were run on subgroups. While evaluating the 1990 reform in Austria, which extended the duration of paid parental leave from 12 to 24 months, Danzer and Lavy^30^ observed that the PISA scores of adolescents of highly educated mothers were significantly and positively impacted. Likewise, Liu and Skans,^24^ who analyzed the extension of paid parental leave from 12 to 15 months in 1988 in Sweden, showed that the school grades and national test results of adolescents of well-educated mothers were significantly improved. Both papers speculated on the institutional setting’s role in explaining the results. Danzer and Lavy emphasized the absence of formal day care centres, suggesting that informal care from grandparents might not surpass well-educated mothers’ maternal care. Liu and Skans noted that heavily subsidized, publicly provided day care was prevalent in 1980s Sweden. They argued that the care provided by low-educated mothers may not be superior in terms of human capital accumulation compared with the public childcare system.

Turning to the papers concluding with mixed results, Dustmann and Schönberg^26^ analyzed a series of reforms extending paid maternity leave in Germany. They observed that the 4-month extensions of paid leave provided first in 1979 and later in 1986 did not generate any significant effects on track choice and average years of schooling. Yet a further extension in 1992, which offered an additional 18 months of unpaid leave, significantly harmed the track choice of adolescents in the long run. Cools et al.^28^ also found mixed effects as they analyzed the impact of the 1992 reform extending paid parental leave and the 1993 reform introducing paternal quota in Norway. While both reforms extended paid leave by three additional weeks that parents could share freely, the 1993 reform also introduced an extra 4 weeks of paternal quota. Whereas the former reform failed to produce significant results, the paternal quota improved the exam scores taken at the end of secondary education. This positive impact was larger for adolescents whose fathers have at least the same level of education as their mothers. These findings underscore the importance of tailoring mandated paternal leave policies. It suggests that policies emphasizing active paternal involvement, particularly when fathers are equipped with a similar educational background to mothers, can contribute to enhanced educational outcomes in the long term.

Turning to the studies which identified positive and significant results, Carneiro et al.^27^ reported that in the long term, Norway’s introduction of paid maternal leave in 1977 decreased the high school dropout rates and increased college attendance significantly. Further, the estimates showed that the positive effects are stronger for adolescents from less educated households, females, and younger siblings. Authors speculated that maternal care is necessarily better than the care provided by grandparents or other informal channels, as they have been the alternative day care arrangements prevalent during 1970s Norway. Ginja et al.’s study^22^ exploiting the 1986 reform in Sweden, which changed the Speed Premium rule of the paid parental leave system, also reported a positive effect on GPA in 9th grade and college attendance by 24 years of age. Further, they saw that the positive impact is more robust for younger siblings and individuals of mothers from higher socioeconomic status. Following the same line of argument, the authors pointed toward the scarcity of formal childcare in 1980s Sweden. They speculated that maternal care is essentially better than any other form of informal care.

### Labour market

We identified three studies that analyzed the long-term impact of parenting-related leave on labour market outcomes during young adulthood using a quasi-experimental design.^21^^,^^26^^,^^27^ Two papers exploited the extension of existing paid maternity leave policies,^21^^,^^26^ one focused on the introduction of paid maternity leave accompanied by the extension of unpaid leave.^27^ The overall results demonstrate mixed evidence (Table 3).

Turning first to the papers which failed to detect any significant effect, the previously mentioned paper by Ahammer et al.^21^ took the labour market outcomes as a secondary long-term well-being indicator, namely the employment rates, occupation, and wages. The treated and untreated individuals have statistically indistinguishable labour market outcomes during young adulthood. Another paper that did not find any significant effect is Dustmann and Schönberg’s^26^ analysis of the impact of Germany’s 1979 reform, which extended paid maternity leave from 2 to 6 months, on wage disparities by implementing the difference-in-differences method.

Turning to the only paper that found a significant impact, Carneiro et al.^27^ exploited the maternity leave reform in Norway in 1977, which introduced paid maternity leave and increased unpaid leave. The reform provided mothers with 18 weeks of paid leave and an additional 1 year of unpaid leave. Unlike previous papers, this legislation was peculiar since it was an introduction rather than an extension. The results from regression discontinuity and differences-in-difference estimates suggested that the reform significantly increased earnings at age 30 by 5 and 7 percentage points, respectively. The positive impact was stronger for males compared with females, for younger siblings compared with firstborns, and for individuals from more educated households. The authors speculated that mothers’ increased time investment mainly drove the positive results. Accordingly, during the 1970s in Norway, the alternative day care arrangements were care provided by grandparents or low-quality informal care; thus, the reform replaced such care with superior care provided by the mother.

## Discussion

Our review yields five key findings. First, the introduction and expansion of paid parenting-related leave policies are associated with improved well-being during adolescence and young adulthood. Second, while extending lengthy leaves may not consistently yield significant long-term outcomes, the introduction of paid leave policies, particularly gender-specific ones such as maternity leaves or paternal quotas, shows evidence of statistically significant benefits. Thirdly, though not statistically tested, the main channel of impact is argued to be parents’ increased time investment during early ages rather than the policy’s financial impact on parents.

Fourth, parenting-related leave policies moderate the influence of maternal socioeconomic status and education on long-term outcomes. This suggests that while these policies aim to promote family well-being, extended leaves may inadvertently worsen societal inequality, highlighting the need for a nuanced understanding of how these policies impact different groups. Fifth, though not tested statistically, institutional factors, including formal day care availability and quality, may be crucial, particularly when considering the interaction between maternal and institutional background. For instance, regions with high-quality subsidized day care raise questions about potential disadvantages when children spend more time with mothers with limited formal education.

Our systematic review has limitations. We exclusively analyzed quasi-experimental studies to assess causal impact, although quantitative and qualitative studies contribute valuable insights. We could not conduct a meta-analysis due to the limited number of papers and significant heterogeneity in outcome measurement, interventions, and institutional contexts.

We identified several limitations in existing studies. Firstly, most papers focus on educational outcomes, with limited attention to labour market and health outcomes, while deviant behaviour remains unexplored. Secondly, outcome variable choices are not always ideal; using hospital admissions as a health indicator is a crude measure. Thirdly, due to quasi-experimental designs, authors estimated the intention-to-treat impact, meaning the estimated effect irrespective of leave utilization. Additionally, studies delve into the main effects, yet incorporating moderating effects that take the maternal education into consideration is likely to produce more nuanced comprehension of policy outcomes. Further, the selection of women into the labour market constitutes a methodological drawback for the quasi-experimental designs. Lastly, authors should delve deeper into the age-specific impacts of parenting-related leave policies, particularly examining why the effects observed during childhood may not persist or be as pronounced during adolescence or young adulthood.

Notwithstanding these limitations, our study has key strengths. Firstly, it is the first systematic review focusing on parenting-related leave’s long-term impact on well-being during adolescence and young adulthood. Secondly, an exclusive focus on quasi-experimental studies presents an overall assessment of causal insights, which is crucial because, due to practical and ethical constraints, it is impractical to assess leave policies through experimental designs.

Our review underscores specific research gaps warranting further exploration. Firstly, our findings underscore the complexity of parenting-related leave policies and their impact in long term, with important implications for policy design. While the introduction of such policies carries possible advantages, they also harbour the potential to inadvertently perpetuate or exacerbate societal inequalities, particularly dependent on varying maternal education and institutional backgrounds. Therefore, further literature should acknowledge that policies do not exert uniform effects across the entire spectrum of education and income distributions. Therefore, researchers should prioritize the conduct of more comprehensive methodological assessments that differentiate between main and moderating effects.

Secondly, researchers can contribute by addressing a broader range of mechanisms and outcomes. Exploring under-researched areas, such as mental health and socially risky behaviours in adolescence, could provide valuable insights, as well as investigate how these policies impact subsequent fertility and marital stability. Most studies focus on maternity leave and prioritize mothers' roles within parental leave, but there is a need to better understand the impact of paternal leave. Some studies primarily view fathers as economic contributors compensating for reduced income. Nevertheless, evidence indicates that gender-specific policies, such as paternal quotas, can exert a substantial impact on adolescent outcomes. Therefore, it is essential to reassess fathers' roles beyond economics and explore the broader social and developmental advantages of policies that encourage active paternal involvement. Thirdly, with emphasis on Nordic countries, most research is done on social-democratic or conservative welfare states. Exploring the impact in liberal welfare settings, such as the UK and USA, would offer a different perspective. Lastly, while the time spent at home is a key channel of impact in all studies, it remains a ‘black box’. Some studies use maternal education as a proxy for the quality of time spent at home, but longitudinal or diary-based time-use data could provide more detailed insights.

In conclusion, the long-term effects of parenting-related leave on well-being during adolescence and young adulthood depend on the duration, institutional context, and baseline conditions. Although quasi-experimental research is limited and yields mixed results, the introduction of paid leave policies, particularly in countries with no or minimal benefits, offers clear advantages. Evidently, this has policy implications for countries without a national leave policy or offering only short durations of paid leave, such as the USA.

## Supplementary Material

ckad228_Supplementary_Data

## Data Availability

The data underlying this article are available in the article and in its online supplementary material.
Key points We systematically reviewed studies which adopted a quasi-experimental design to analyze the impact of parenting-related leave policies on well-being during adolescence and young adulthood, specifically on health, education, and labour market outcomes. The quasi-experimental research exploring the long-term effects of parenting-related leave policies is relatively limited and predominantly focused on maternity leaves. Moreover, a notable imbalance exists with an overemphasis on their influence on educational outcomes. The existing empirical evidence suggests that the expansion of already long parenting-related leave durations may not consistently yield statistically significant outcomes. Yet, the introduction of leave policies or gender-specific policies, such as paternal quotas, has been associated with notable and statistically significant benefits. These results underscore significant policy implications for countries that lack a comprehensive national leave policy or offer only short durations of paid leave, such as the USA.
