# Supplementary material for: Long-term impact of parenting-related leave policies on adolescents’ well-being: a systematic review of quasi-experiments
Source: Eur J Public Health. 2024 Jan 9;34(2):272–82. doi: 10.1093/eurpub/ckad228 (PMC10990559; doi:10.1093/eurpub/ckad228)
Supplement: ckad228_Supplementary_Data [file ckad228_supplementary_data.docx]

**SUPPLEMENTARY MATERIAL 1**

**PICOS for inclusion and exclusion**

| **Criteria** | **Inclusion** | **Exclusion** |
| --- | --- | --- |
| Participants (Population) | Children of women and men who were entitled to take leave after the policy intervention. | Populations of nations located outside Europe (excluding Turkey and Russia), Northern America (Canada and USA), and Australia. |
| Interventions | Legislated policies introducing or extending leaves that allow employees to take time off work due to pregnancy or taking care of a new born or newly adopted child. | Medical or sick-leave policies. |
| Control | Children born before the legislation of the intervention under analysis. |  |
| Outcomes | The following outcomes of children when they are above 12 years of age:   1. Education: educational attainment, grade completion, track choice, GPA, PISA scores, exam grades, etc. 2. Physical and mental health: hospital admissions, chronic diseases, obesity, subjective wellbeing, mental disorders, etc. 3. Labour market outcomes: earnings, occupation, employment status, etc. 4. Deviant behaviour: Crime propensity, alcohol abuse, addiction, gun use, etc. |  |
| Study design | Quasi-experiments: difference-in-differences, regression discontinuity design, instrumental variable design, synthetic controls, and any combination of these. | Reviews and observational studies that do not use the strategies mentioned for causal identification. |

**SUPPLEMENTARY MATERIAL 2**

We applied the following keyword variants for each search term to the title, abstract, and keywords section of the articles:

- Parental leave: paid parental leave, paid leave, parental leave, maternity leave, maternal leave, paternity leave, paternal leave
- Children: child, children, teenager, teenagers, adolescent, adolescents, minor, minor, youth, preteen, preteens, teen, teens, juvenile, juveniles
- Wellbeing outcomes:
  - Education: education, school, class, college, diploma, academic, performance, achievement, grade, drop out, literacy
  - For mental and physical health: health, physical wellbeing, medical, disability, chronic illness, obesity, diabetes, development, mental health, mental disorder, depression, anxiety, happiness, mental wellbeing, emotional wellbeing
  - Labour market outcomes: employment, unemployment, labor, labour, wage, income, earning, occupation, poverty
  - Deviant behaviour: alcohol, drug, substance abuse, crime, violence, gun
- Quasi-experimental design: natural experiment, quasi-experiment, regression discontinuity, RDD, difference in difference, DID, DD, instrumental variable, IV, synthetic control

This yielded a final search string of:

(“paid parental leave” OR “paid leave” OR “parental leave” OR “maternity leave” OR “maternal leave” OR “paternity leave” OR “paternal leave”) AND (“child” OR “children” OR “teenager” OR “teenagers” OR “adolescent” OR “adolescents” OR “minor” OR “minors” OR “youth” OR “preteen” OR “preteens” OR “teen” OR “teens” OR “juvenile” OR “juveniles”) AND ((“education” OR “school” OR “class” OR “college” OR “diploma” OR “academic” OR “performance” OR “achievement” OR “grade” OR “drop out” OR “literacy”) OR (“health” OR “physical wellbeing” OR “medical” OR “disability” OR “chronic illness” OR “obesity” OR “diabetes” OR “development” OR “mental health” OR “mental disorder” OR “depression” OR “anxiety” OR “happiness” OR “mental wellbeing” OR “emotional wellbeing”) OR (“employment” OR “unemployment” OR “labor” OR “labour” OR “wage” OR “income” OR “earning” OR “occupation” OR “poverty”) OR (“alcohol” OR “drug” OR “substance abuse” OR “crime” OR “violence” OR “gun”)) AND (“natural experiment” OR “quasi-experiment” OR “regression discontinuity” OR “RDD” OR “difference in difference” OR “DID” OR “DD” OR “instrumental variable” OR “IV” OR “synthetic control”)
